# Chronic granulomatous disease associated with Duchenne muscular dystrophy caused by Xp21.1 contiguous gene deletion syndrome: Case report and literature review

**DOI:** 10.3389/fgene.2022.970204

**Published:** 2023-01-13

**Authors:** Shaohua Bi, Liying Dai, Liangliang Jiang, Lili Wang, Mia Teng, Guanghui Liu, Ru-Jeng Teng

**Affiliations:** ^1^ Division of Neonatology, Anhui Provincial Children’s Hospital, Hefei, Anhui, China; ^2^ Department of Pediatrics, Anhui Provincial Children’s Hospital, Hefei, Anhui, China; ^3^ First Affiliated Hospital of Anhui Medical University, Hefei, Anhui, China; ^4^ Department of Pediatrics, Medical College of Wisconsin, Milwaukee, WI, United States

**Keywords:** chronic granulomatous disease, Duchenne muscular dystrophy, contiguous gene deletion syndrome, neonatal sepsis, hypertriglyceridemia

## Abstract

Chronic granulomatous disease (CGD) and Duchenne muscular dystrophy (DMD) are X-linked recessive disorders whose genes are 4.47 Mb apart within Xp21.1. A combination of both diseases is rare with only five cases reported in the literature where it is known as Xp21.1 “contiguous gene deletion syndrome”. We describe a male neonate who presented with sepsis at 19 days of age. The diagnosis of CGD with DMD was established through copy number variation sequencing (CNV-seq) with an extensive 7.5 Mb deletion of Xp21.2-Xp11.4 of the proband. One of his elder sisters and his mother are carriers. The deletion includes six known genes: glycerol kinase (*GK*), dystrophin (*DMD*), cilia- and flagella-associated protein 47 (*CFAP47*), gp91 (*CYBB*), Kell antigen (*XK*), and retinitis pigmentosa GTPase regulator (*RPGR*). Laboratory assays revealed an increased creatine kinase (CK) level, decreased gp91 expression, and a positive nitroblue tetrazolium test. Due to the extensive gene deletion and the poor prognosis, the family determined to pursue conservative management without further laboratory workup. The patient passed away from a fulminant infection at the age of three-month at a local medical facility. To the best of our knowledge, this case of Xp21.1 contiguous gene deletion syndrome represents the most extensive deletion of genes in this region ever reported. A literature review of similar cases is presented.

## Introduction

Chronic granulomatous disease (CGD) is a rare primary immune deficiency caused by a defective nicotinamide adenine dinucleotide phosphate (NADPH) oxidase complex. NADPH oxidase is composed of five subunits (gp91phox, p22phox, p47phox, p67phox, p40phox) and the absence of any one subunit can cause the disease (Chiriaco et al., 2016). CGD patients suffer from repeated bacterial and fungal infections due to their phagocytes’ inability to generate superoxide through respiratory burst (Warren and Shimada, 2018). CGD’s most common genetic defect is a point mutation in the CYBB gene. Most reported CGD cases had either a base mutation or an intragenic gene deletion (Roos et al., 2021). However, results from sequencing determinations of copy number variations (CNVs) uncovered a few cases identified as having Xp21.1 contiguous deletion syndrome. The clinical manifestations of patients with the Xp21 deletion spectrum can be quite variable. McLeod syndrome (MLS) may coexist if the deletion involves the *XK* gene due to its proximity. MLS patients have XK antigen deficiency, spinous erythrocytosis, and a specific nervous system phenotype (Roulis et al., 2018). Although the *DMD* gene is far from the *CYBB* gene, DMD may also be part of the spectrum if the deletion is extensive enough.

Duchenne muscular dystrophy/Becker muscular dystrophy (DMD/BMD) is one of the most common X-linked recessive genetic muscular diseases, and it also has a poor prognosis. DMD was named for the French neurologist, Guillaume Duchenne (Duchenne, 1867), and it is quite rare with an incidence of about 1 in 3,500 live male births. Typical clinical features of DMD include progressive muscular atrophy, pseudohypertrophy of the gastrocnemius muscle, and a highly elevated blood level of creatine kinase (CK), 50-300 times the normal levels in the early stage of the disease and slowly decreasing with age (Kim et al., 2017). Classical DMD affects mainly adolescent males, who generally lose the ability to stand and walk before their teens. DMD patients often die by their 20s from either heart failure or respiratory failure. This disease is rarely diagnosed in neonates without a targeted genetic test.

Here, we report a neonatal case of CGD combined with DMD diagnosed through a genetic workup. The neonate was transferred to our facility with a severe infection. Flow cytometry showed decreased gp91phox expression. Low-depth whole-genome sequencing of copy number variations (CNV-Seq) showed a ∼7.5 Mb deletion in the loci between 21.1 and 11.4 on the short-arm of the X chromosome (Xp). The genetic data established the diagnosis of DMD before the typical increase in the muscle enzyme in the blood and the various disorders that only manifest later in life.

## Case presentation

**FIGURE 1:**
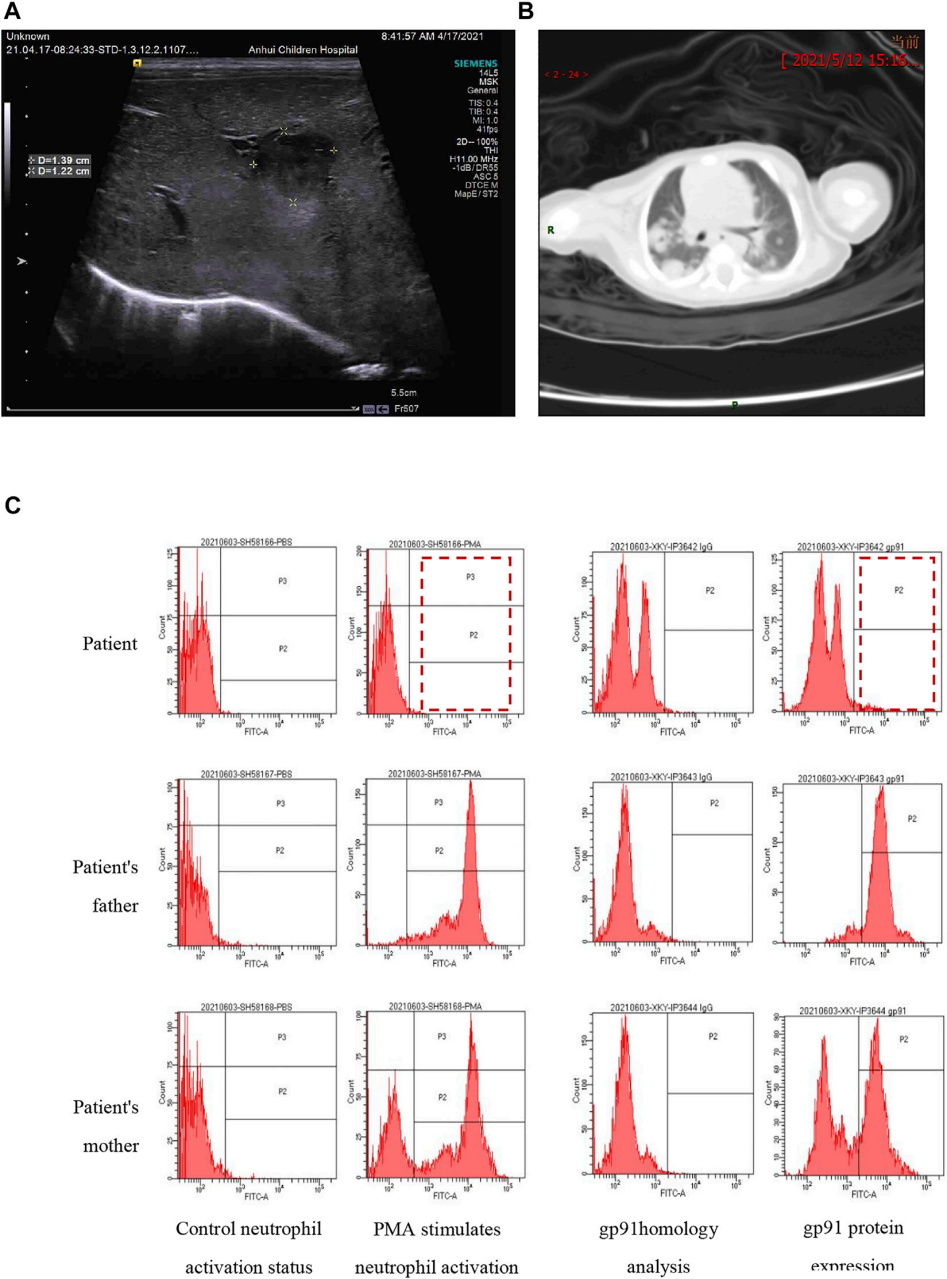
The images show suspected liver abscesses **(A)** and lung abscesses **(B)**. The neutrophil respiratory burst test **(C)** was performed by flow cytometry. The left panel shows neutrophil activation by phorbol 12-myristate 13-acetate (PMA). The father had normal activation with a single peak in zone P2 and P3 (>90% activation), while the proband had no activation (1.5%), and the mother had two activation peaks (62.7%). The right panel shows gp91phox expression: 4.6% in the proband, 51.7% in the mother, and >90% in the father. The results indicate that the proband is a case of CGD while his mother is a carrier.

The 19-day-old male neonate was born to a G5P3023 mother via cesarean section due to macrosomia at a gestation of 41^+1^ weeks with a birth weight of 4250 g. Apgar scores were 10 for both 1 and 5 min. After 3 days of broad-spectrum antibiotic treatment, the neonate was transferred to our hospital under the impression of neonatal sepsis without improvement. The blood culture from the referring hospital did not identify the pathogen. Physical examination upon admission showed a rectal temperature of 38.1°C, but the patient appropriately responded to stimulation. He had a soft and flat anterior fontanelle, mild tachypnea with clear air entry, a heart rate of 145 beats/min, no hepatosplenomegaly, full range of motion of all four limbs, and normoactive bowel sound. A laboratory test showed a white blood cell count of 33.24 × 10^9^/L with 71% neutrophils, a hemoglobin of 143 g/L, platelet count of 322 × 10^9^/L, and C-reactive protein (CRP) of 133.84 mg/L. Biochemical assays showed an alanine aminotransferase (ALT) of 124.2 U/L, aspartate aminotransferase (AST) of 137.5 U/L, and an abnormally high triglyceride level of 8.66 mmol/L (0.68–1.69 mmol/L). The blood culture upon admission was also negative, probably due to antibiotic treatment before the transfer. The creatine kinase (CK) level was 1115 IU/L (0–210 IU/L) which was high, but typical for DMD patients, who usually have a CK above 2,000 IU/L. Abdominal ultrasound demonstrated several hypoechoic areas within the liver compatible with liver abscess. The two large foci were measured as 2.5 cm × 2.5 cm x 2.0 cm at 3.5 cm below the rib and 1.4 cm × 1.3 cm x 1.2 cm at 3.8 cm below the xiphoid process (Figure 1A). Chest CT showed multiple nodules in both lung fields with cavity formation compatible with lung abscess (Figure 1B). The neutrophil respiratory burst test and the quantitative gp91^phox^ protein flow cytometry confirmed the diagnosis of CGD (Figure 1C). No abnormality was found in echocardiography or eye examination.

Informed consent for genetic testing was obtained from both parents, and the study was conducted according to the declaration of Helsinki and approved by the medical ethics committee of Anhui Children’s Hospital. CNV-seq was used for its faster turnaround time, availability, and reasonable cost in our region. Blood was obtained from the proband and his family through venipuncture. Genomic DNA was extracted from mononuclear white cells using a human genomic DNA extraction kit (Qiagen, Hilden, Germany). DNA was extracted from K_2_-EDTA-treated venous blood using the TIANamp blood DNA kit (#DP348-03). The integrity of the extracted DNA was evaluated by electrophoresis on 2% agarose. DNA specimens with OD 260/280 between 1.8 and 2.0, concentrations above 20 ng/μL, and amounts >500 ng were used to prepare the DNA library with a Hieff NGS^®^ OnePot DNA Library Prep Kit for Illumina^®^ (#12205ES24, Yeasen Biotechnology, Shanghai, PRC), according to the provided protocol. Magnetic beads were used to purify the amplified PCR products before Nextseq500 sequencing using the SE150 mode. The readouts in FASTQ format that passed the quality assurance test were then compared to reference data and aligned using Bowtie 2 into BAM files. The Picard tools were employed to obtain the mapping ratio to remove the repeated sequences generated by PCR. The processed sequences were used for analysis by the CNV kit with the pipeline for the BAM files according to the predetermined bin size. Gene fragments with normal chromosome number and size less than 100 KB were used to compare against available databases. The total sequencing data amounted to 1.03 Gb, with 7,815,225 reads, and an average genome sequencing depth of about 0.3x. Mutations were identified by comparison with available databases (dbSNP at www.ncbi.nlm.nih.gov/snp/; ExAC at www.exac.broadinstitute.org/; 1000 genomes at www.1000genomes.org/) and performing a hazard prediction analysis on the reliable variation spectrum by filtering out invalid mutations (SIFT at https://sift.bii.a-star.edu.sg/; PolyPhen-2 at http://genetics.bwh.harvard.edu/pph2/; MutationTaster at www.mutationtaster.org/). The mutations were rated according to the ACMG pathogenicity criteria (Richards et al., 2015). The software detected the off-line data, analyzed the genes contained in the CNV interval, judged the microdeletions/duplications in the sample data, and associated them with the DECIPHER database (www.decipher.sanger.ac.uk/), dbVar database (www.bigd.big.ac.cn/databasecommons), DGV database (http://dgv.tcag.ca/dgv/app/home), ClinGen (https://www.clinicalgenome.org/), OMIM database (www.omim.org), and other public databases.

**FIGURE 2:**
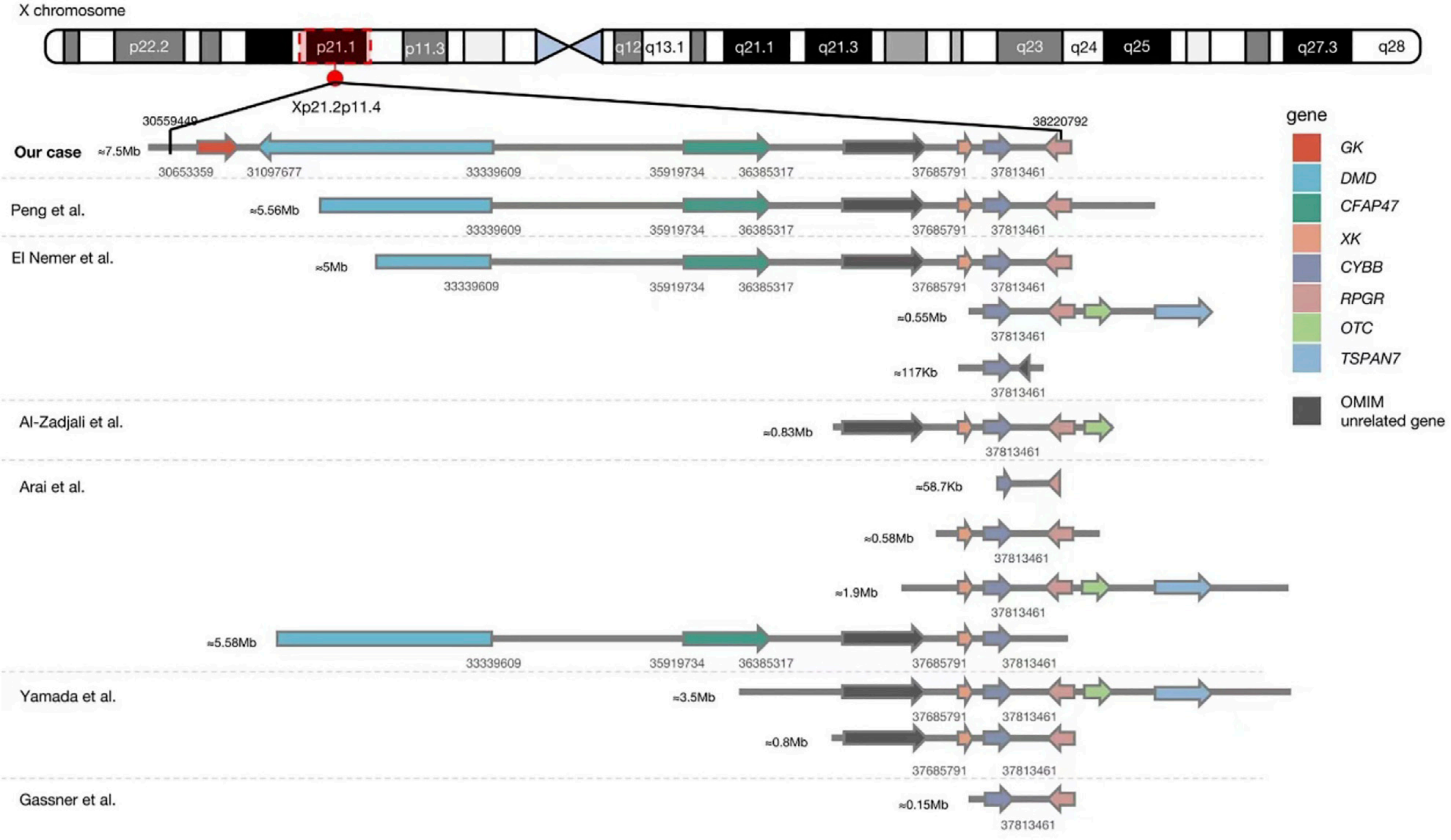
Comparison between our case and previously reported Xp21.1 contiguous gene deletion syndrome with known deletion sites. The detailed genetic data of eight cases in one case series reported by Lhomme et al. (Lhomme et al., 2020) (8/8 with McLeod phenotype, 2/8 with DMD, and 1/4 with retinitis pigmentosa) are unavailable, so they cannot be aligned in this figure. Our patient had the most extensive gene deletion of ∼7.5 Mb involving six genes with known phenotypes and a gene of unknown significance.

**FIGURE 3:**
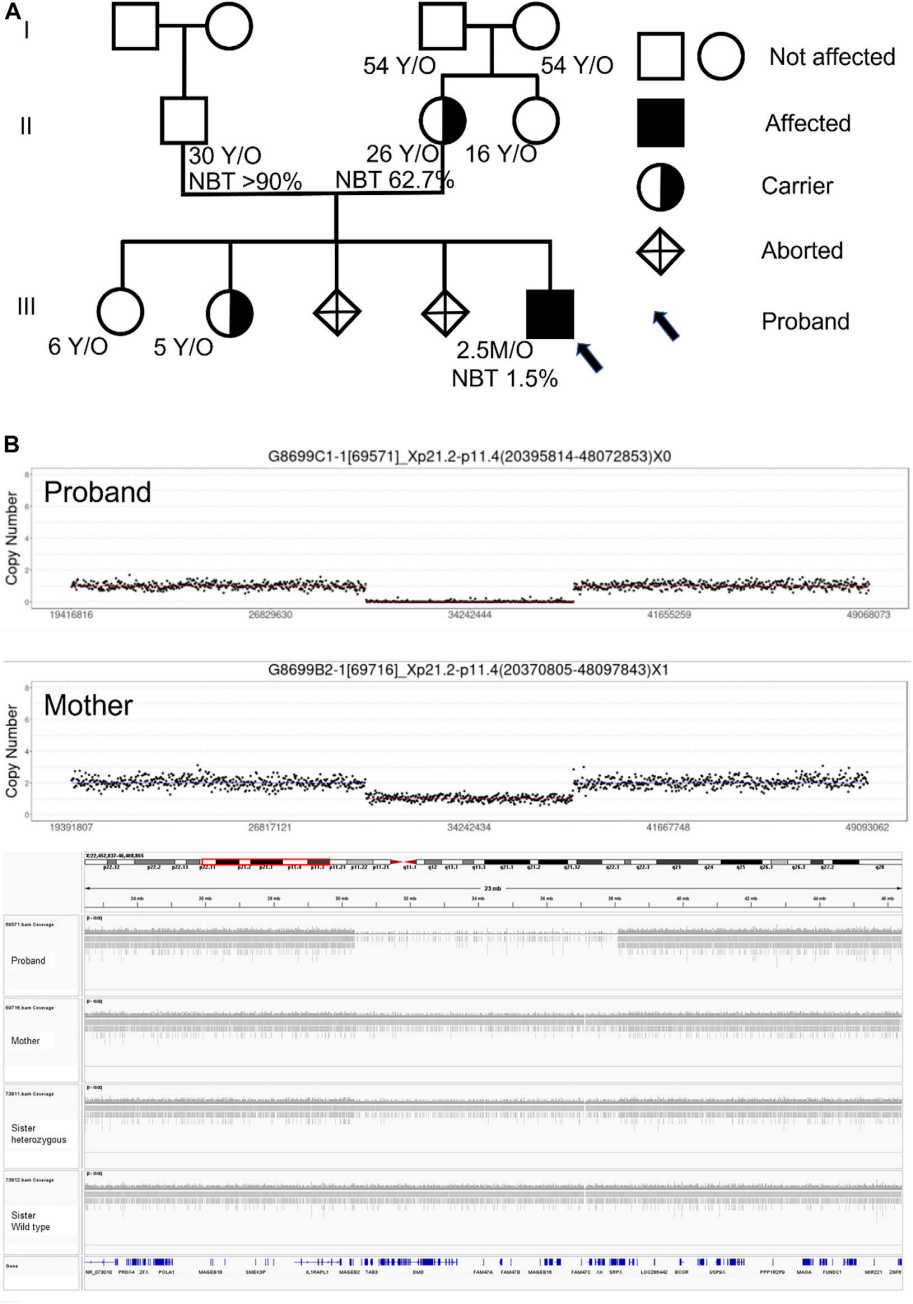
**(A)** Pedigree, nitroblue tetrazolium test (NBT), and genetic test results of family members. All four grandparents and the father have no Xp21.2-11.4 deletion, but the mother and one older sibling are carriers. **(B)** CNV-seq results of the proband and his mother.

The genetic analysis showed the deletion of a 7.5 Mb fragment of chromosome Xp21 between p21.2 and p11.4 (Chr X: 30577566-38080045, SEQ [grch37] del Xp21.2-p11.4) in the proband (Figure 2). One of his older sisters and his mother were heterozygous for the same deletion (Figure 3). The DECIPHER database contains many pathogenic CNVs within this region of the X-chromosome, including six OMIM disease entities such as the *GK* gene that is associated with glycerol kinase deficiency (*GKD*, OMIM #307030); the *DMD* gene that is associated with DMD (*DMD*, OMIM #310200); the *CFAP47* gene that is associated with X-linked spermatogenic failure (*SPGFX3*, OMIM#301059); the *XK* gene that is associated with McLeod syndrome with or without CGD (*MLS,* OMIM #300842); the *CYBB* gene that is associated with X-linked CGD (*CGDX*, OMIM #306400); and the *RPGR* gene that is associated with retinitis pigmentosa (RP3, OMIM#312610).

After admission, the neonate was treated empirically with broad-spectrum antibiotics. His body temperature stabilized after 1 day of treatment, and the blood assay results and CRP gradually normalized. The liver abscess resolved after two weeks of antibiotics based on ultrasound imaging. Chest CT revealed that the multiple nodules in both lungs associated with lung abscesses were also decreased in size after antibiotics. The infant was discharged home two months later. After an extensive multidisciplinary care conference with the family, the decision was made to pursue conservative medical management due to the anticipated poor long-term outcome. The family refused further workup to confirm the other disorders associated with the extensively deleted chromosome. Unfortunately, the infant passed away 1 month after discharge at a local medical facility.

## Discussion

We report a rare case of Xp21.1 contiguous gene deletion syndrome (Chr X: 30577566-38080045; SEQ [grch37] del Xp21.2-p11.4), confirmed by CNV-seq, presenting as a neonatal infection involving both liver and lungs. The mother and one of the older sisters are heterozygous for the same deletion. The 7.5 Mb deletion is the most extensive compared to the reported cases (Table 1). A positive respiratory burst test, a reduced gp91^phox^ expression, and genetic sequencing confirmed the diagnosis of CGD. The atypical elevation in serum CK level in a genetically confirmed case of DMD can be explained by the limited muscle damage early in life and indicates that the borderline CK level is not a useful biomarker during early infancy to diagnose DMD.

**TABLE 1:** Age of onset, initial clinical manifestation, and genetic information of reported cases.

Reported	Age of onset	Initial clinical manifestations	Inheritance	Genes involved in the report	Deletion site	Deletion size	References
Our case	19 D/O	Neonatal infection, liver and lung abscess	Inherited	GK, **DMD**, CFAP47, XK, CYBB, RPGR	Xp21.2-Xp11.4	∼7.5 Mb	-
Gassner et al	10Y/O	pulmonary aspergillosis	*De novo*	CYBB, RPGR	N/A	∼0.15 Mb	15
Lhomme et al	1.2 Y/O	Pneumonia	*De novo*	XK, CYBB	Xp21.1-Xp21.4	210 Kb	17
	3.9 Y/O	Pneumonia	Inherited	XK, CYBB	N/A	N/A	
	1 M/O	Adenitis	*De novo*	XK, CYBB	N/A	N/A	
	6 M/O	Adenitis	Inherited	**DMD**, XK, CYBB	Xp21, complete	5.5 Mb	
	1.2 Y/O	Liver abscess	*De novo*	XK, CYBB	N/A	3.75 Mb	
	2 M/O	Adenitis	Inherited	XK, CYBB	N/A	N/A	
	2.6 Y/O	Adenitis	Inherited	XK, CYBB	N/A	N/A	
	1.5 Y/O	Osteomyelitis	Inherited	XK, CYBB	N/A	1.3 Mb	
Peng et al	4M/O	Skin infection	Inherited	**DMD**, CFAP47, XK, CYBB, RPGR	N/A	∼5.56 Mb	22
El Nemer et al	N/A	N/A	N/A	**DMD**, CFAP47, XK, CYBB, RPGR	N/A	∼5 Mb	23
	N/A	N/A	N/A	CYBB, RPGR, OTC, TSPAN7	N/A	∼0.55 Mb	
	N/A	N/A	N/A	CYBB	N/A	117 Kb	
Al-Zadjali et al	10Y/O	Pyoderma, chest infections, gluteal abscess, bowel granuloma	Inherited	XK, CYBB, RPGR, OTC	N/A	∼0.83 Mb	24
Arai et al	N/A	N/A	N/A	CYBB, RPGR	N/A	58.7 Kb	25
	N/A	N/A	N/A	XK, CYBB, RPGR	N/A	∼0.59 Mb	
	N/A	N/A	N/A	XK, CYBB, RPGR, OTC, TSPAN7	N/A	∼1.94 Mb	
	N/A	N/A	N/A	**DMD**, CFAP47, XK, CYBB	N/A	∼5.71 Mb	
Yamada et al	3Y/O	Perianal abscesses, lymphadenitis	*De novo*	XK, CYBB, RPGR, OTC, TSPAN7	N/A	∼3.5 Mb	26
	20Y/O	Perianal abscesses	Inherited	XK, CYBB, RPGR	N/A	0.8 Mb	

N/A: not described in the reports.

CGD is a rare immune deficiency disorder caused by an inability to generate superoxide anion due to defective NADPH oxidase activity (NOX2). CGD patients are prone to repeated bacterial infections with typical pathogens, including *Staphylococcus aureus*, *Burkholderia cepacia*, *Aspergillus* spp, and *Serratia marcescens* (Segal et al., 2000; Winkelstein et al., 2000; Dekkers et al., 2008). Most CGD patients are diagnosed after 1 year (Hou et al., 2019). The main clinical manifestations are repeated and severe organ infections, including lungs, lymph nodes, liver, and skin. Occasionally CGD can present as a cardiac abscess or phlebitis, complicating the diagnosis, especially in neonates (Song et al., 2014). The most common type of CGD (∼66%) is caused by a mutation on Xp21 involving the *CYBB* gene. According to the literature, the pathology in less than 1% of CGD patients is caused by the contiguous gene deletion syndrome (Roos et al., 2021). The database of our region is not available for comparison.

Compared with simple CGD, the deletion of larger fragments around the *CYBB* gene can lead to a more complex phenotype. DMD is a neuromuscular disease caused by the deletion or mutation of the *DMD* gene on Xp21 (Maggio et al., 2016) and usually presents after childhood with patients developing respiratory failure or heart failure by the age of 20. There is no clear clinical manifestation in DMD neonates, so the diagnosis is rarely established during this period. Although the CPK level was abnormally high in our case, it was not typical for DMD, in which the CPK level is usually above 2,000 at diagnosis. The lack of muscle damage can explain the lack of clinical manifestation of DMD early in life and why the CPK level was not in the DMD range in our case. We could not confirm the diagnosis of DMD without performing the genetic test.

Our case had four other known genes deleted. The most commonly associated *XK* gene deletion in males may lead to MLS presenting as a slowly progressive disorder that primarily involves the central/peripheral nervous system, heart, and immune system, mimicking symptoms of Huntington’s disease, accompanied by cognitive decline and mental disorders. The average age of onset of the MLS is between 30 and 40 years (Jung et al., 2007; Gassner et al., 2017). The *XK* gene encodes a complex Kell blood group on the erythrocyte membrane resulting in echinocytosis and hemolysis (Yu et al., 2001; Lhomme et al., 2020). Due to the proximity of genes, some MLS patients can present with CGD, chronic hemolysis, or neuromuscular symptoms after infancy (Roulis et al., 2018; Lhomme et al., 2020).

Pseudo-hypertriglyceridemia secondary to high glycerol blood levels has been described in *GK* deletion (Zaffanello et al., 2004). One of the manifestations of *GK* deletion is a high glycerol level in urine which may help establish the diagnosis (Wikiera et al., 2021). Cryptorchidism and strabismus are two of the most common clinical signs of *GK* deletion, which were not seen in our case. Our case had hypertriglyceridemia, but we did not have the opportunity to verify whether it was due to high glycerol levels in the blood.

The *RPGR* deletion has been reported as one type of retinitis pigmentosa (Cehajic-Kapetanovic et al., 2020). The proximity of the *RPGR* genes and the *CYBB* gene makes it a common co-existing disorder (11/16) (El Nemer et al., 2000; Peng et al., 2007; Yamada et al., 2010; Arai et al., 2012; Al-Zadjali et al., 2015; Gassner et al., 2017; Lhomme et al., 2020). In our case, X-linked retinitis pigmentosa, cone-rod dystrophy, and primary ciliary dyskinesia secondary to the *RPGR* gene mutation may manifest later in life. The *CFAP47* deletion can cause male infertility (Liu et al., 2021). Although these problems will not be present during infancy, they are critical for genetic counseling.

Our literature search has identified only seven reports of Xp21.1 contiguous gene deletion syndrome, including twenty patients with a deletion involving the *CYBB* gene (El Nemer et al., 2000; Peng et al., 2007; Yamada et al., 2010; Arai et al., 2012; Al-Zadjali et al., 2015; Gassner et al., 2017; Lhomme et al., 2020). The age of clinical presentation, initial clinical manifestation, and genetic information of the reported cases are summarized in Table 1. The lack of proper molecular technology prevented us from identifying cases before the year 2000. The range of chromosome deletion was between 58.7 Kb and 5.56Mb, with an average of 2.22 Mb (Figure 2). It is worth noting that the distance between the *CYBB* and *XK* genes is only 47 Kb which explains why 80% of reported patients (16/20) had both *CYBB* and *XK* gene deletions. Although some patients did not show neurological problems, almost all had hematological changes due to erythrocyte Kell blood group antigen reduction. The distance between *CYBB* and *DMD* genes is about 4.47Mb, so only six cases (including ours) had both manifestations. Without the genetic test, we would not have been able to detect the co-existence of DMD in our CGD patient, who was brought to us during his early infancy.

It is crucial to pursue an extensive genetic analysis, if possible, to detect other gene deletions in the Xp21 segment in neonatal patients with CGD (Lhomme et al., 2020). This is especially critical because the future male offspring have a 50% chance of being affected, while 50% of the female offspring will be carriers with variable degrees of presentation. The whole family genetic screening showed that the mother was the one with the *de novo* mutation, and one of the older sisters was identified as a carrier (Figure 3). The neighboring genes cover many complex phenotypes that will not manifest early in life. However, their involvement can be challenging for long-term management and genetic counseling. Other than the initial presentation as CGD, knowledge of the associated problems like muscular dystrophy, hemolytic risk, hypertriglyceridemia, blindness, and long-term neurological morbidities is essential for the family to make the appropriate decisions. Although bone marrow transplantation is a promising treatment for CGD (Lhomme et al., 2020), the extensive gene deletion in our case painted a grim picture for the family and influenced the decision whether or not to pursue aggressive management.

In conclusion, we reported a newborn patient with CGD complicated with multiple disorders due to an extensive deletion involving Xp21.2-Xp11.4. The ∼7.5 MB fragment deletion is the most extensive ever reported. The co-existence of CGD and glycerol kinase deficiency has not been reported in the literature. We understand the limitation of using CNV-seq to identify the genetic changes in our index case, but the availability and the short turnaround time led us to adopt this method. The death of the baby in early infancy prevented us from observing other clinical manifestations, and this case report emphasizes the importance of genetic analysis in patients with early onset CGD.

## Data Availability

The datasets for this article are not publicly available due to concerns regarding participant/patient anonymity. Requests to access the datasets should be directed to the corresponding author.
